# Bilateral vocal cords paralysis requiring urgent tracheostomy on COVID-19 patient: a case report

**DOI:** 10.1186/s40981-022-00578-5

**Published:** 2022-10-26

**Authors:** Kazuya Omura, Kiyoyasu Kurahashi

**Affiliations:** Department of Anesthesiology and Intensive Care Medicine, International University of Health and Welfare Narita Hospital, 852 Hatakeda, Narita, Chiba, Japan

**Keywords:** COVID-19, Tracheostomy, Vocal cord paralysis, Laryngeal edema, Infection control

## Abstract

**Background:**

SARS-CoV-2 infection has many manifestations, including otolaryngological symptoms.

**Case presentation:**

A 60-year-old man with severe dyspnea underwent endotracheal intubation followed by 68 h of mechanical ventilation. After extubation, he left the ICU without any significant complications. Four days after the extubation, he developed dyspnea, which deteriorated the next 2 days, and stridor became evident. A fiberoptic laryngoscope revealed bilateral vocal cord edema and paralysis, which required an emergency airway. We decided to perform an awake tracheostomy under local anesthesia while considering protection for airborne infection to healthcare providers. The tracheostomy was closed when the edema and paralysis of the vocal cords were ameliorated.

**Conclusions:**

A COVID-19 patient who underwent injurious ventilation developed vocal cord paralysis and edema 6 days after extubation, leading to an emergency tracheostomy. Close attention to the upper airway of COVID-19 patients is essential since the pathophysiology of the present incident may be specific to the viral infection.

## Background

COVID-19, caused by SARS-CoV-2 infection, has exhibited various clinical manifestations. A recent systematic review reported that the primary symptoms in COVID-19 patients were fever (58.7%), cough (54.5%), malaise (29.8%), dyspnea (30.8%), and fatigue (28.2%) [1]. In addition, otolaryngological symptoms, such as sore throat, hoarseness, and dysphagia, are also reported as manifestations of COVID-19 [2]. Some reported dysphonia and vocal cord damage related to COVID-19 [2–6]; however, the mechanisms are not fully elucidated.

We reported a case of severe COVID-19 patient with bilateral vocal cord paralysis one week after extubation and required an emergency tracheostomy.

## Case presentation

**Fig. 1:**
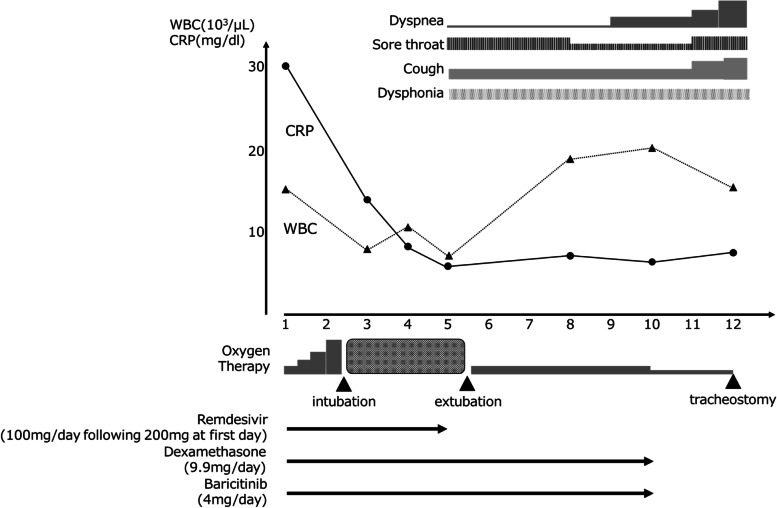
The course after hospitalization. WBC, white blood cell; CRP, C-reactive protein

**Fig. 2:**
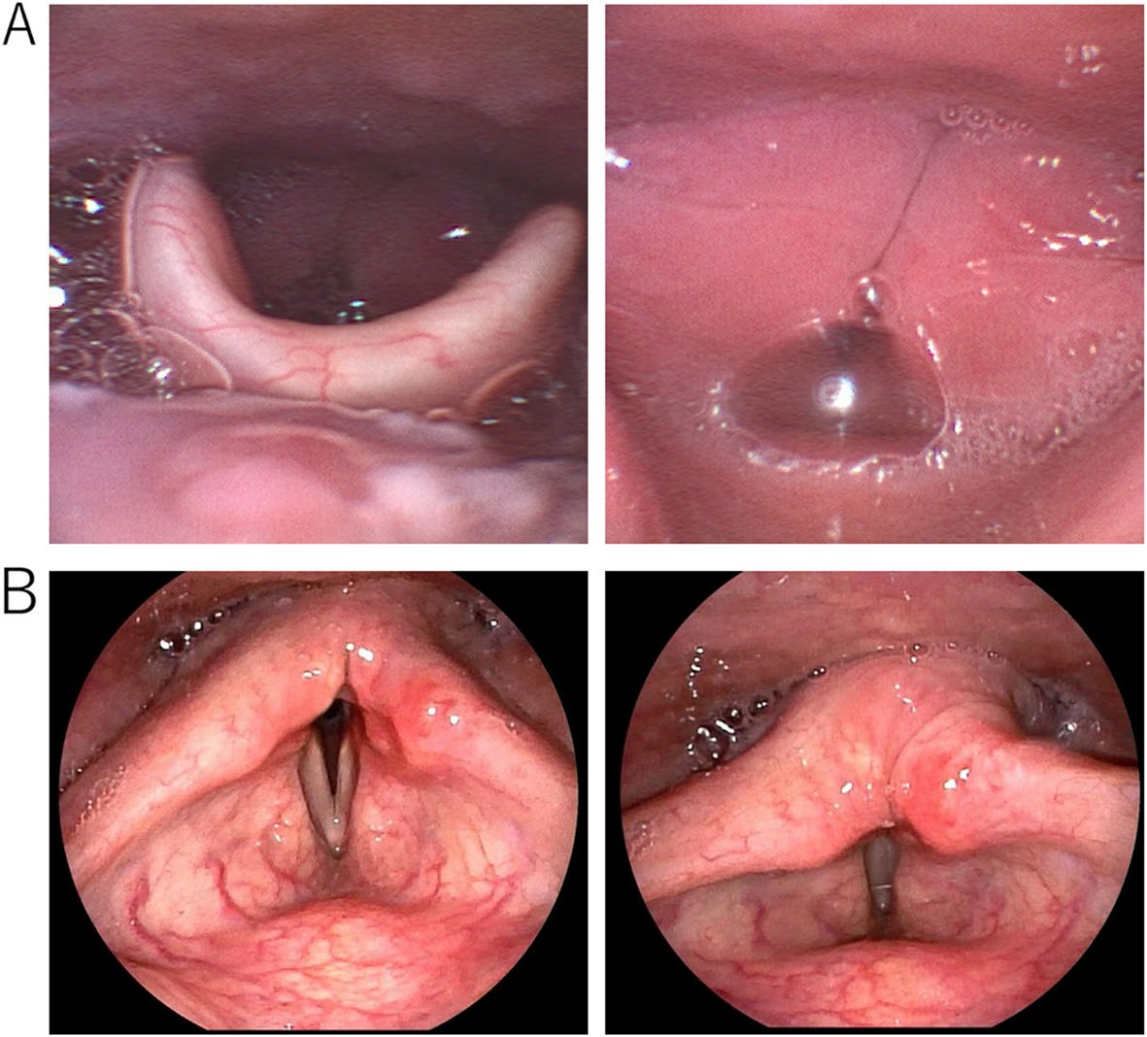
The finding of a pharyngeal fiberscope. **A** Six days after the extubation. **B** Six days after the tracheostomy

A 60-year-old man with a height of 162.5 cm and a weight of 70.6 kg, who has a medical history of bronchial asthma, medicated with inhaling steroids, presented with a fever. Three days later, the patient was diagnosed as COVID-19 with PCR positive for SARS-CoV-2. He was admitted to our hospital with a high fever and loss of appetite 7 days after the diagnosis. On admission, his consciousness was clear, body temperature 36.8 °C, SpO2 94%. Chest CT images showed ground glass opacity spread over a quarter of both lung areas. We initiated treatments with remdesivir 100mg/day following 200 mg on the first day and dexamethasone 9.9mg/day. The course after hospitalization is shown in Fig. 1. On the second day of hospitalization, the patient experienced desaturation, and tracheal intubation was performed uneventfully. During the mechanical ventilation, the patient was deeply sedated with propofol, midazolam, and fentanyl. The cuff pressure of the tracheal tube was controlled between 25 and 30 cmH_2_O with an automated cuff pressure controller. After 68 h of mechanical ventilation, the tracheal tube was removed without performing a cuff leak test due to the relatively short duration of intubation. There were no findings suggestive of upper airway obstruction or asthma attack, but weak vocalizations were present, and the patient complained of mild dysphonia. A modified water swallowing test revealed no swallowing problem. He had a persistent sore throat, hoarseness, and dry cough and complained of dyspnea, especially at night, 4 days after extubation. Six days after the extubation, the patient complained of severe dyspnea, and biphasic stridor was heard. We performed a pharyngeal fiberscope and found bilateral vocal cord edema and laryngeal midline paralysis (Fig. 2A). Initially, an emergency tracheal intubation was considered; however, we decided to perform an awake tracheostomy under local anesthesia to avoid the risk of cannot ventilate cannot intubate (CVCI). A significant improvement in his vocal cord’s movement was observed with oral corticosteroid therapy with hydrocortisone 300 mg for 2 days, then decreased by 100 mg every 2 days for a total of 6 days. Although there are restrictions on the glottis opening (Fig. 2B), the tracheostomy was closed 14 days after the tracheostomy without further respiratory complications.

## Discussion

We report a patient with severe COVID-19 who required an emergency tracheostomy due to bilateral vocal cord paralysis a week after extubation.

Many factors can cause vocal cord paralysis, and tracheal intubation is one of the significant factors. The causes of vocal cord paralysis include compression of the recurrent laryngeal nerve by the tracheal tube cuff and direct damage to the vocal cords during intubation. The frequency of paralysis is about 0.1% in patients who undergo tracheal intubation for surgery, and most are unilateral [7]. It usually occurs within 36 h after extubation [8]. Long-term intubation is one of the risk factors for vocal cord paralysis. Compared with the intubation duration of 3 h or less, the incidence increases two- and fifteen-fold when intubated for 3–6 h and 6 h or longer, respectively [7]. Among the episodes of thousands of and millions of COVID-19 patients around the globe, there are several reports on vocal cord paralysis [3–6] (Table 1). It occurred between 3 and 17 days after the extubation. Patients who did not undergo tracheal intubation also experienced vocal cord paralysis. In the present case, dexamethasone was discontinued 2 days before tracheostomy, which may have exacerbated vocal cord edema. However, bilateral vocal cord paralysis and the fact that the symptoms and signs of airway obstruction became evident a few days after the extubation does not suggest that mechanical damage to vocal cords or recurrent nerves during intubation was the cause of the paralysis. Instead, the virus or concomitant inflammation is highly suspected as one of the elements of the incident. Further studies on these topics are warranted.

**Table 1:** Reports on vocal cord paralysis related to COVID-19

Author	Age	Sex	Duration of ventilation (days)	Part of palsy	Clinical symptom	Timing of diagnosis	Treatment
Curros [3]	74	Male	11	Bilateral	Cough at night dyspnea	35 days after discharge	Elective tracheotomy
Korkmaz [4]	57	Female	No intubation	Left	Hoarseness	15th day of the onset	Laryngoplasty
Eltelety [5]	21	Male	No intubation	Right	Hoarseness	7th day of the onset	Conservative therapy
Jungbauer [6]	74	Female	17	Bilateral	Dyspnea	2 weeks after discharge	Laryngoplasty
Omura (current case)	61	Male	3	Bilateral	Cough at night dyspnea	19th day of the onset (6 days after extubation)	Emergency tracheotomy

An emergency tracheal intubation is the most common procedure for impending airway obstruction. An emergency tracheostomy or cricothyroid ligament incision may be the best option when CVCI is predicted in such patients, like in the present case.

Aerosol exposure to healthcare providers during airway management is a concern during the SARS-CoV-2 pandemic. Tracheostomy is a procedure with a high risk of aerosol infection. A Slovenian recommendation described a rapid cricothyrotomy under deep sedation and deep muscle relaxation without tracheal intubation on COVID-19 patients [9]; however, the best procedure may vary case by case. We chose tracheostomy under local anesthesia in the present case. Even if the situation can be an emergency, careful considerations should be addressed: arrange a negative-pressured OR, control traffic between ICU and OR while the patient is transferred, the skilled operator should be recruited, such as otolaryngologists, qualified anesthesiologists and nurses should be involved, enforce personal protective equipment (PPE), obtain additional staffs and equipment in case of an emergency during the procedure, and secure the way to communicate between the OR and the control room.

## Conclusion

A patient with COVID-19, intubated for 4 days, had bilateral vocal cord paralysis and developed rapid and intense laryngeal edema a week after extubation. We should weigh the patient’s condition, such as the degree of the airway emergency and the CICV risks, against environmental issues, including airborne exposure to healthcare providers. Although the exact mechanism is unclear, there exists vocal cords palsy and edema in COVID-19 patients long after the extubation or even without a history of tracheal intubation.

## Data Availability

Not applicable.
